# Made-to-measure malaria vector control strategies: rational design based on insecticide properties and coverage of blood resources for mosquitoes

**DOI:** 10.1186/1475-2875-13-146

**Published:** 2014-04-16

**Authors:** Gerry F Killeen, Aklilu Seyoum, John E Gimnig, Jennifer C Stevenson, Christopher J Drakeley, Nakul Chitnis

**Affiliations:** 1 Ifakara Health Institute, Environmental Health and Ecological Sciences Thematic Group, Ifakara, Kilombero, Morogoro, United Republic of Tanzania; 2 Liverpool School of Tropical Medicine, Vector Biology Department, Pembroke Place, Liverpool L3 5QA, UK; 3 Division of Parasitic Diseases and Malaria, Centers for Disease Control and Prevention, Atlanta, Georgia 30333, USA; 4 Department of Immunology and Infection, Faculty of Infectious and Tropical Diseases, London School of Hygiene and Tropical Medicine, Keppel Street, London WC1E 7HT, UK; 5 Johns Hopkins Malaria Research Institute, Johns Hopkins Bloomberg School of Public Health, Baltimore, MD 21205, USA; 6 Department of Epidemiology and Public Health, Swiss Tropical and Public Health Institute, Basel, Switzerland; 7 University of Basel, Basel, Switzerland; 8 Fogarty International Center, National Institutes of Health, Bethesda, MD 20892, USA

**Keywords:** *Plasmodium*, *Anopheles*, Vector control, Mosquito, Malaria, Target product profile

## Abstract

Eliminating malaria from highly endemic settings will require unprecedented levels of vector control. To suppress mosquito populations, vector control products targeting their blood hosts must attain high biological coverage of all available sources, rather than merely high demographic coverage of a targeted resource subset, such as humans while asleep indoors. Beyond defining biological coverage in a measurable way, the proportion of blood meals obtained from humans and the proportion of bites upon unprotected humans occurring indoors also suggest optimal target product profiles for delivering insecticides to humans or livestock. For vectors that feed only occasionally upon humans, preferred animal hosts may be optimal targets for mosquito-toxic insecticides, and vapour-phase insecticides optimized to maximize repellency, rather than toxicity, may be ideal for directly protecting people against indoor and outdoor exposure. However, for vectors that primarily feed upon people, repellent vapour-phase insecticides may be inferior to toxic ones and may undermine the impact of contact insecticides applied to human sleeping spaces, houses or clothing if combined in the same time and place. These concepts are also applicable to other mosquito-borne anthroponoses so that diverse target species could be simultaneously controlled with integrated vector management programmes. Measurements of these two crucial mosquito behavioural parameters should now be integrated into programmatically funded, longitudinal, national-scale entomological monitoring systems to inform selection of available technologies and investment in developing new ones.

## Background

While anti-parasitic drugs and vaccines will be essential for the final stages of malaria elimination, their effectiveness as transmission control interventions will rely heavily upon first achieving unprecedented levels of vector control in settings with historically high levels of endemicity [1-4]. The most important malaria parasites of humans are entirely dependent on people as their only secondary, mammalian hosts, so the most potent vector mosquito species are those with highly specialized behaviour adapted to feeding upon humans indoors at night when they are asleep [5-9]. Thus, the majority of the most potent vectors distributed across the tropics predominantly feed upon humans inside houses, where they can be effectively controlled and even eliminated with long-lasting insecticidal nets (LLINs) or indoor residual spraying (IRS) [6-9]. While LLINs and IRS can reduce transmission by these human-specialized, indoor-feeding mosquito species by as much as two orders of magnitude, there are many parts of Africa and the Pacific where malaria transmission can occur at levels four orders of magnitude greater than that required to sustain the parasite population [4]. Much of the residual transmission that persists following scale up of LLINs and/or IRS is sustained by mosquitoes that can evade contact with these insecticidal interventions by feeding upon humans and animals outdoors [4,6-9]. It will therefore not be possible to eliminate malaria transmission from most of the tropics without developing additional scalable vector control strategies which complement LLINs and IRS by extending intervention coverage of the blood resources that mosquitoes depend upon beyond humans and their houses [4,6-10].

To achieve this laudable goal in practice, product developers, manufacturers and end-users need a manageably short list of ecologically-defined target product profiles to work towards that are based on field-measured behavioural and physiological traits of wild vector populations [11]. From the resulting arsenal of complementary vector control products, malaria control programmes will need to select the most effective subset of these options, based on national or regional surveys of these same key behavioural and physiological traits [8,12]. While quite a long list of underlying parameters of mosquito, parasite and human populations determine the overall level of malaria transmission that occurs in a locality, many of these are difficult or impossible to measure routinely across nationally representative scales and relatively few of them are direct targets of vector control measures [11]. Here, a simple conceptual framework based on mathematical models is described that allows new and existing tools for controlling adult malaria vectors to be prioritized and optimized for specific contexts, by predicting their relative merits based on only two field-measurable behavioural parameters of local mosquito populations and two field-measurable indicators of how those mosquitoes interact with specific vector control products.

### Biological coverage of all blood resources available to mosquitoes

Suppression of mosquito populations with vector control products depends on high biological coverage [13], broadly defined as the proportion of all available sources of blood that is effectively modified to kill, deter, contaminate, or incapacitate mosquitoes at times and places when they attempt to use it. The crucial difference between conventional *demographic coverage* of humans with a protective measure, and *biological coverage* of blood resources that mosquitoes depend upon, is that the latter is inclusive of *all* forms of that resource, while the former is merely the *subset* of that resource that humans represent at the times and places when they can use the intervention. While this definition can be expanded and applied to any resource mosquitoes may exploit (Killeen GF, Seyoum A, Gimnig JE, Corliss G, Kiware SS, Stevenson JC, Drakeley CJ, Chitnis N, personal communication), host attack and blood acquisition are the best understood of all resource utilization behaviours and can be conveniently, passively surveyed by attracting vectors to hosts sampled from within quantifiable populations of humans or animals [10]. These behaviours are also the most obvious and common target for vector control interventions, because they are obligate behaviours for all *Anopheles* and determine the rate of pathogen transmission [10]. Biological coverage of all available blood resources with a protective measure (*C*_*A,p*_) can therefore be estimated as the product of *demographic coverage*, defined and surveyed as the proportion of humans protected on a given night (*C*_*h*_), and two field-measurable mosquito behavioural parameters: the human blood index (*Q*_*h*_) and the proportion of human exposure that occurs indoors (*π*_*h,i*_) [13]:

(1) $$C_{A,p}=\pi_{h,i}Q_{h}C_{h}$$

Beyond defining coverage of vector control interventions in a measurable and biologically meaningful way, field measurements of these two behavioural parameters can also guide the specification of ideal target product profiles for delivering insecticides to humans or livestock [9,13-16].

### Blood source as a determinant of intervention selection and impact

**Figure 1:**
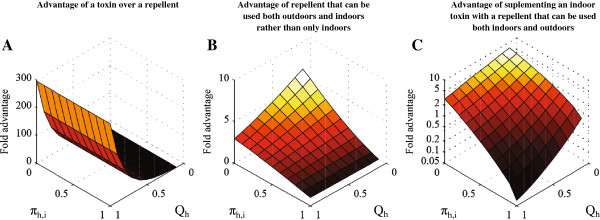
**Simulated predictions of the comparative transmission control advantages (>one-fold) and disadvantages (<one-fold) of specific target product profiles, and combinations thereof, as a function of the baseline proportion of human exposure to vector bites occurring indoors (*π***_***h,i***_**) and the baseline proportion of blood meals obtained from humans by the vector population (*Q***_***h***_**).** In all simulated scenarios [14-16], high demographic coverage (*C*_*h*_ = 0.8) is assumed for personal protection products with **A**: toxic *versus* repellent properties used both indoors and outdoors; **B**: repellent properties that can be used indoors and outdoors *versus* indoors alone; and, **C**: repellent properties that can be used indoors and outdoors combined with an exclusively indoor-applicable toxic product *versus* the exclusively indoor toxic product alone. In all scenarios, all toxicity is assumed to act on contact before mosquitoes feed so that products with toxic (*θ*_*μ,pre*_ =0.8, *θ*_*μ,post*_ =0) and repellent (*θ*_*Δ*_ =0.8) profiles confer equivalent personal protection (*ρ* = 0.8) and differ only in the level of community-level protection achieved [14-16].

Human blood indices are difficult to measure where vector populations are sparse or primarily rest outdoors, and are inevitably prone to bias arising from heterogeneities of sampling efficiency by resting site category [17,18]. Nevertheless, such estimates are remarkably useful as predictors of large-scale variations of pre-existing malaria transmission intensity [19] and are equally important for selecting optimal vector control methods (Figure 1). The vast majority of human malaria infections are caused by *Plasmodium falciparum* and *Plasmodium vivax,* which are both strict anthroponoses, so the most efficient vectors in the world are those which predominantly feed upon humans [19]. Fortunately, this dependence upon human blood also renders them vulnerable to population control [7,8] and even elimination [9] with high coverage of people with insecticidal personal protection measures such as LLINs and IRS. The more a vector depends upon human blood, the greater will be the impact of human personal protection measures upon their population density, longevity and transmission potential, and the greater will be the advantage of pesticides which kill rather than repel mosquitoes (Figure 1A) [13,16,20]. For highly efficient, anthropophagic and endophagic vectors that are most readily controlled with indoor use of contact toxins, it is predicted that outdoor repellent use confers no advantage (Figure 1B) and indoor repellent use dramatically undermines the otherwise massive impact of LLIN use (Figure 1C) [15,20].

However, approximately 40% of all *P. falciparum* infections [21] and 95% of *P. vivax* infections [22] occur outside of sub-Saharan Africa, where diverse primary vectors [23] predominantly feed on animals rather than humans [19]. Where human blood is unimportant to vector survival and reproduction, personal protection of people will have negligible impact upon the mean density, longevity or stability of those mosquito populations but may achieve community-level protection of non-users by simply blocking vector contact with infectious users and *vice versa*[13,16]. It is therefore irrelevant whether that is achieved through toxicity or repellency (Figure 1B) so personal protection against highly zoophagic vectors should be maximized by whichever mode of action is most practical [16]. Zoophagic vectors usually prefer to feed outdoors where vapour-phase insecticides should have significant advantages because enclosing structures to provide physical protection and application surfaces for solid-phase residual toxins are typically absent, impractical or even undesirable [15]. By definition, any repellent action of an insecticide is manifested at lower, sub-lethal doses of insecticides than those required to kill mosquitoes [24,25] so the former non-lethal mode of action should be optimized to maximize the personal protection afforded by a vapour-phase active ingredient against vectors that primarily feed upon animals.

**Figure 2:**
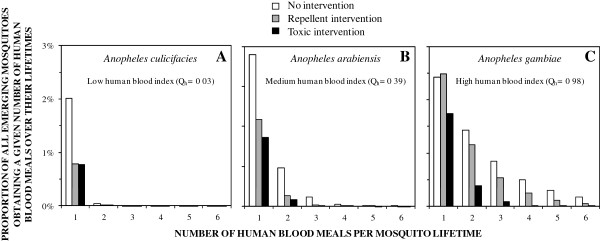
**Simulated predictions of the proportion of emerging mosquitoes that will take a given number of blood meals from humans over their lifetimes, depending on their natural preference for humans and the protection of those humans with interventions that either repel or kill them, taking *Anopheles culicifacies* (A), *An. arabiensis* (B) and *An. gambiae* (C) as examples of vectors with weak, intermediate and strong preferences for feeding on humans, respectively.** All simulations were implemented exactly as described previously [14], assuming that these mosquitoes differ only in their preferences for human and cattle hosts (parameterized as per [16]), and that high demographic coverage (*C*_*h*_ = 0.8) and protective efficacy (*ρ* = 0.8) of the intervention measures are maintained at all times of the day (*π*_*h*_ = 1). All toxicity is assumed to act on contact before mosquitoes feed so that products with toxic (*θ*_*μ,pre*_ =0.8, *θ*_*μ,post*_ =0) and repellent (*θ*_*Δ*_ =0.8) profiles confer equivalent personal protection (*ρ* = 0.8) and differ only in the level of community-level protection achieved [14-16]. The proportional frequency of emerging mosquitoes which take a given number of human blood meals per lifetime (*F*_*i*_) is calculated as product of the mean probability of survival per feeding cycle (*p*_*f*_) and the human blood index (*Q*_*h*_) to the power of the number of blood meals (*i*) divided by the sum of the values for this term for all possible numbers of blood meals: $F_{i}={\left(p_{f}Q_{h}\right)}^{i}/\sum_{i=0}^{\infty }{\left(p_{f}Q_{h}\right)}^{i}$. Parameter values for the relative availability of humans, compared to cattle, were estimated based on published field observations of variations in human blood index with local host abundance, exactly as previously described for *Anopheles gambiae* and *Anopheles arabiensis*[27], and by direct comparison of observed attack rates upon cattle and humans for *Anopheles culicifacies*[28].

Toxic insecticides may therefore have substantive advantages over repellents for targeting humans where people represent an important blood source to mosquitoes but not where they primarily rely upon animal blood. This potential advantage of contact toxins over repellents, and its dependence upon the host preferences of the vector, is illustrated in terms of human feeds per mosquito lifetime in Figure 2. For a mosquito such as *Anopheles culicifacies*, which rarely feeds on human blood but does so often enough to act as a primary vector [16,26], a repellent should achieve community-level suppression of malaria transmission that is equivalent to that of a toxic product conferring the same level of personal protection. This is because feeding upon humans is a relatively rare event, so most transmission is mediated by mosquitoes taking the bare minimum of two human blood meals required to complete the transmission cycle. Mosquitoes that survive after being repelled from a human or human household have a very low chance of ever feeding on another human. High coverage of efficacious repellents can therefore break the transmission cycle by making the possibility of a mosquito feeding on humans twice even more remote, so the epidemiological impact of this mode of action is equivalent to killing mosquitoes outright with toxicants (Figure 2A). However, for a mosquito with a strong or even moderate preference for human blood, such as *Anopheles gambiae* and *Anopheles arabiensis,* respectively, diversion away from a protected human user and extension of host-seeking activity undoubtedly increases associated mortality risks, but many will survive and feed on other humans nearby so the toxic product always has a considerable advantage (Figure 2B and Figure 2C) that is very much needed when faced with the massive transmission levels they mediate [2,4,16].

**Figure 3:**
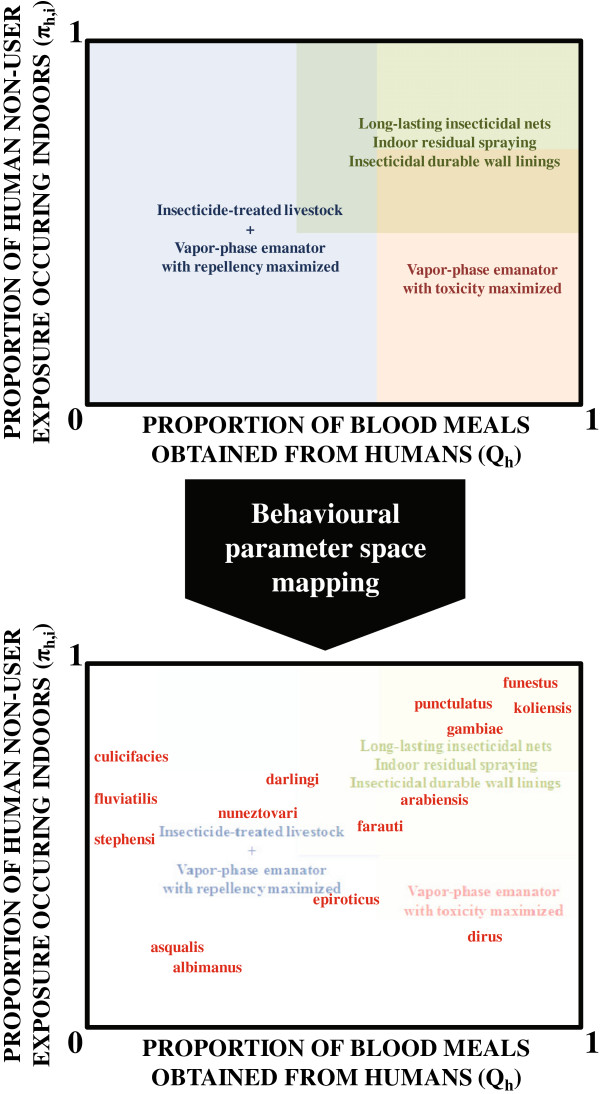
A conceptual illustration of how optimal vector control interventions and intervention combination could be mapped across vector behaviour parameter space, populated by field measurements of diverse target vectors.

Beyond directly protecting their occasional human victims, mosquito-toxic insecticides may also be applied to livestock, to enable population control of zoophagic vectors and achieve greater proportional reductions of transmission where these are the preferred hosts for dominant local vectors [29-31], and transmission is fundamentally easier to manage because zoophagic mosquitoes are less efficient vectors of anthroponotic *Plasmodium*[16-19]. However, many important vector species in residual transmission systems, such as *An. arabiensis* in Africa, *Anopheles darlingi* in Latin America and *Anopheles farauti* in the Pacific, can readily feed upon either humans or animals [6-8], so that they represent quite efficient vectors requiring a combination of complementary measures to achieve effective intervention coverage of all preferred host types (Figure 3). Furthermore, they express both zoophagy and anthropophagy with remarkable phenotypic plasticity, resulting in spectacular variation in human blood indices across very fine scales [17,18,27,32], so these vectors alone may exhibit behavioural properties encompassing large tracts of the parameter space and associated intervention needs represented by Figure 3.

Current strategic frameworks for developing new vector control tools emphasize the importance of overall human biting rates, expressed using classical Macdonald-Ross models as the product of mosquito population density per human (*m*) and the square of the human biting frequency per mosquito (*a*) [11]. While both parameters are of central importance to baseline levels of transmission, and therefore to the levels of control that will be required to eliminate it, the human-feeding frequency has far greater influence on local transmission intensity, and therefore geographic distribution of malaria risk [19], because each transmission event requires two blood meals upon humans so vectorial capacity is approximately proportional to its square (*a*^*2*^) [33]. The human biting frequency is also proportional to the human blood index (*a* = *Q*_*h*_/*f* where *f* is the mean duration of the feeding cycle length of individual mosquitoes) and is therefore far more relevant to intervention prioritization and optimization (Figures 1, 2 and 3). For example, it is difficult to envisage a situation in which LLINs or IRS would be de-prioritized as the first-choice options for tackling anthropophagic *An. gambiae* or *Anopheles funestus*, regardless of their population density.

While human biting frequency (*a*), human blood index (*Q*_*h*_) or an equivalent term feature in essentially all process-explicit models of malaria transmission [33,34], and are of central importance to selecting and optimizing the most appropriate vector control strategy (Figures 1, 2 and 3), the classical modelling studies that underpinned planning of the GMEP [35,36] not only ignored the way in which frequent feeding upon animals (*a* or *Q*_*h*_ → 0) attenuates the population suppression effects of human-targeted vector control measures [13,16], these models also omitted parameters to account for the fact that mosquitoes feeding upon humans may not necessarily do so where and when they can be targeted with LLINs or IRS [2,6-8,10,12,13,15,37].

### Maximizing protective coverage of humans and houses

Feeding upon alternative hosts is by no means the only form of behavioural resilience or resistance [4,8] that limits biological coverage of personal protection measures such as LLINs: some mosquitoes attack humans at times and places where these measures are not realistically applicable. For any product conferring personal protection against mosquito bites, estimating the proportion of human exposure to mosquito bites that occurs at times when it is practical to use it (*π*_*h*_) is critically important to measuring the maximum amount of protection that can be realistically expected [13,38,39]. In the case of LLINs, this definition can be approximated as the proportion of normal exposure to mosquito bites upon humans lacking LLINs which occurs indoors (*π*_*h,i*_) or during sleeping hours (*π*_*h,s*_) when it would be practical to use a net [38,39]. These parameters are measured in the field by weighting the observed indoor and outdoor biting rates at each period of the night by the surveyed mean proportion of humans that are indoors and outdoors, respectively, at that time [5,38,39]. While these parameters can be measured for individual people, or strata within human populations [5,40], it is their community-wide mean values as experienced by the mosquito population that determines the magnitude of the mass effect of vector control interventions [2,16,37]. In Africa, consistently high values for this key behavioural parameter, even in some settings with long-established high coverage of LLINs, are primarily driven by the preference of *An. gambiae*, *An. arabiensis* and *An. funestus* for feeding at times when most people are indoors asleep, rather than any strong or consistent preference for feeding indoors *per se*[38]. These estimates of the proportion of human exposure occurring inside houses can only be applied to indoor interventions against host-seeking mosquitoes such as an LLIN, but do help illustrate the conceptual basis of this parameter as the upper limit for the *de facto* level of direct personal protection, through immediate toxicity or repellency, that can realistically be expected from using one. Historically, similar indicators of pre-intervention biting times were strong predictors of vector population vulnerability to suppression with IRS [41] and derived estimates of the proportion of exposure occurring indoors for net users (*π*_*h,i,n*_) suggest at least half of residual transmission now occurs outdoors in African settings with high LLIN coverage [4]. Furthermore, the proportion of human exposure to residual vector populations that occurs indoors has recently dropped in some settings with high coverage of LLINs or IRS [6-8,12]. It has been suggested that these altered patterns of mosquito activity can be explained by the persistence of hungry mosquitoes until people are unprotected at dusk and dawn, a form of behavioural phenotypic plasticity that can be classified as behavioural *resilience* rather than *resistance*[7,8]. However, recent modelling analysis suggests shifting distributions of the times when wild mosquito populations actually feed successfully upon human communities using LLINs should not be manifested in the biting rates experienced by unprotected human volunteers because they are fully exposed [42]. Changes in biting patterns observed by human landing catch may therefore represent genuine emergence of behavioural resistance in the form of altered innate feeding time preferences [6,42].

Regardless of whether observations of outdoor-feeding behaviour reflect pre-existing resilience or emerging resistance, it is clear that they will have to be addressed with insecticide-treated clothing [43,44], vapour-phase insecticides that protect humans outside of their houses [24], or some other intervention that prevents bites from outdoor-feeding mosquitoes [42,45]. While repellents may be ideal for protecting against outdoor exposure to zoophagic vectors [13,15,16], some outdoor-feeding Asian species such as *Anopheles dirus*[46] and *An. farauti*[47,48] often feed predominantly upon humans [7] so vapour-phase insecticides that lack repellent properties may be preferred to maximize toxic exposure, mortality rates and population suppression of these species (Figure 3). Furthermore, the persistence and even predominance of indoor-feeding behaviours in vector populations exposed to high coverage of LLINs and/or IRS [4,5,38,48,49] suggests there is still considerable room for improvement upon these technologies for killing mosquitoes that enter houses [42,50]. The proportions of human exposure which occur indoors and outdoors are therefore important and dynamic indicators of vector behaviour that malaria control programmes should survey on a routine basis [6,8,12,13,38] so that they can manage malaria transmission in the same integrated, evidence-based, locally tailored and adaptive manner as agricultural pests [51].

While these indicators are ideal for LLINs, field measurements of the maximum proportion of human exposure which is directly preventable (π_h_) by other personal protection interventions will require more careful consideration, especially for insecticidal clothing or repellent products with usage patterns that are more difficult to survey because they are portable, used outdoors, or require frequent re-application. The issue of where and when protective measures should be applied becomes particularly important for repellents in settings where zoophagic vectors co-exist with anthropophagic counterparts that have already been suppressed with LLINs or IRS applied indoors, or with insecticide-treated clothing or non-repellent, mosquito-toxic, vapour-phase insecticide emanators applied outdoors. Suppressed populations of such potent vectors, that are otherwise behaviourally vulnerable to control, may well rebound if the toxic action of these products is undermined when they are supplemented with repellents [14,15,20]. It is therefore important to note that while both repellent and toxic products may be required in many scenarios where one or more vectors exhibit intermediate or wide-ranging values of the human blood index (Figure 3), these should not be applied in the same time and place but rather combined in a complementary manner, ideally to achieve a “push-pull” strategy similar to those applied to agricultural pests [52].

### Biological coverage of indoor resting sites *versus* human blood indoors

If one considers interventions which target resources other than blood, that mosquitoes may use several times in a single gonotrophic or feeding cycle, it is clear that existing definitions for *Q*_*h*_ and *π*_*h*_ , based on the concept of protecting humans against exploitation by mosquitoes as sources of blood, must be extended and generalized further. Taking IRS of resting sites in human habitations as the most obvious example, models of malaria transmission and vector population dynamics could be parameterized using estimates of the utilization rate of indoor resting sites, quantified as the mean number of times that a typical mosquito rests indoors per gonotrophic cycle (*α*_*r,i*_, where *α* represents the mean number of times a mosquito utilizes any given resource during a single gonotrophic cycle, *r* represents all resting site resources, and *i* represents the subset of resting sites that are indoors (Killeen GF, Seyoum A, Gimnig JE, Corliss G, Kiware SS, Stevenson JC, Drakeley CJ, Chitnis N, personal communication)). However, despite the widespread use and global prioritization of IRS as a frontline malaria vector control tool [53], the only available field measurements of this parameter are undoubtedly underestimated because they rely upon captures of resting mosquitoes at a single point in time in the early morning. Such a temporal snapshot of resting events will obviously fail to detect mosquitoes that rested on the surveyed indoor surfaces but then left again before they were surveyed. Entomological survey methods for dramatically improving the detection efficiency of resting events clearly need to be developed, presumably by exploiting the diversity of marker systems that are now available for labelling insects [54], or the rapidly improving technologies for observing them visually [55,56].

In the absence of direct measurements of *α*_*r,i*_ , it is possible to use *π*_*h,i*_ as a reasonable surrogate in many contexts, based on the assumption that many vectors which preferentially feed inside houses usually rest there too. Defining vector control coverage in terms of mosquito dependence upon obtaining blood from humans indoors has therefore proven useful for rationalizing the differential impact of not only LLINs, but also IRS, upon sympatric primary vectors in a variety of settings [9,13]. However, using *π*_*h,i*_ as a surrogate for *α*_*r,i*_ does have major limitations and may be very misleading for many vectors with divergent values of these two parameters because they feed indoors but rest outdoors or *vice versa*. Vector species that combine indoor feeding with natural or insecticide-induced outdoor resting are important contributors to persistent residual malaria transmission, despite high coverage of LLINs or IRS [7,14,24,25,57] across Africa [58-60], Asia [46,48] and the Americas [5]. Conversely, Figure 3 suggests that IRS should be only modestly effective against some of the major vectors of southern Asia, such as *An. fluviatilis*, *An. culicifacies* and *An. stephensi* because their most common sibling species and variants obtain only a minor proportion of their blood meals from humans [19] and substantive proportions of these may occur outdoors [26]. However, IRS nevertheless delivers impressive impact against malaria transmission by these vectors [61] because they usually rest inside houses and cattle sheds after they have fed [26]. Indoor resting spaces are the most obvious and important non-blood resources for mosquitoes, are closely associated with human blood, and can be targeted with existing “off-the-shelf” vector technology. It is therefore remarkable that utilization of indoor resting spaces by mosquitoes remains to be quantitatively understood, or fully exploited with vector control using rationally-designed products [42,50].

In theory, the concept of biological coverage outlined here can indeed be extended to enable rational assessment of vector control measures targeting specific subsets of poorly defined resources, including indoor or outdoor resting sites, by measuring the rate at which mosquitoes utilize them per gonotrophic cycle (Killeen GF, Seyoum A, Gimnig JE, Corliss G, Kiware SS, Stevenson JC, Drakeley CJ, Chitnis N, personal communication). It is therefore feasible to map out predicted and observed impacts of IRS, as well as other intervention strategies targeting specific subsets of resting sites, across behavioural parameter space in a similar manner to the way in which Figures 1 and 3 do so for blood resources, which are more readily defined and quantified. However, in practice, the measurement of these parameters is more challenging. Given that several important vector species either feed indoors but rest outdoors, or feed outdoors but rest indoors, lack of adequate procedures for measuring the rates at which mosquitoes utilize subsets (*x*) of resting sites (*r*) that can be targeted with insecticides (*α*_*r,x*_), the most important of which are the indoors surfaces (*x* = *i*) inside houses and other shelters (*α*_*r,i*_), is clearly a methodological deficit that needs to be urgently addressed.

## Conclusions

For mosquito populations to be successfully suppressed, vector control products targeting their blood hosts need to attain high biological coverage of all available sources, rather than merely high demographic coverage of a targeted subset such as humans indoors [9,13]. Beyond defining biological coverage in a quantifiable manner, the human blood index and the proportion of human exposure that occurs indoors may also be used to define optimal target profiles for diverse products and product combinations to protect humans or livestock against blood-seeking mosquitoes (Figures 1, 2 and 3). For vector mosquitoes that feed only occasionally upon humans, preferred animal hosts may be optimal targets for mosquito-toxic insecticides, and vapour-phase insecticides optimized to maximize repellency, rather than toxicity, may be ideal for directly protecting people against indoor and outdoor exposure. However, for vectors that primarily feed upon people, repellent vapour-phase insecticides may be inferior to toxic ones and may undermine the impact of contact insecticides applied to human sleeping spaces, houses or clothing if combined in the same time and place.

The theory of biological coverage may also be extended to other life history parameters, such as indoor resting, to assess the impact of vector control interventions, such as IRS, which target resting mosquitoes. While measurements of the proportion of human blood meals that occur indoors often correlate well with the rates at which vectors utilize indoor resting sites, many important vectors do not rest where they feed. In such cases, the proportion of human blood meals occurring indoors cannot be used a proxy measure of indoor resting site utilization rate, and aspiration capture of resting mosquitoes only gives a snapshot of mosquito distributions at specific points in time, so new entomological methods for detecting *all* resting events at insecticide-targetable surfaces are urgently needed.

The conceptual framework outlined here relates to anthroponotic malaria parasites as specific motivating examples. However, these concepts and strategies should also be directly applicable to other mosquito-borne anthroponoses, such as dengue, urban yellow fever and lymphatic filariasis, or adapted to zoonotic pathogens such as *Plasmodium knowlesi*, Rift Valley fever and West Nile virus, for which their implications should be different but no less rational, so that diverse target species can be simultaneously controlled with integrated vector management programmes [62]. However, rather than stereotyped, hypothetical schematics such as Figure 3, control programmes, policy makers, research funders, and product developers need such maps of vector behaviour parameter space to be populated with real field estimates of these mosquito behaviours and impacts of specific interventions upon those vectors. Only then will they be able to inform the selection of available technologies based on rational expectations of impact, and also prioritize investment in developing new ones. Entomological techniques for measuring these two critical behavioural parameters are well established and have changed little since classic texts were written during, or immediately after, the failed Global Malaria Eradication Programme decades ago [5,17,18,63,64]. Unfortunately, they have only been applied at village or district scales thus far, with inconsistent methodology, and with haphazard distribution across times and locations, because they have been predominantly funded through sporadic, short-term research projects. Fortunately, recent global policy emphasizes strengthening capacity for routine entomological surveillance at Ministries of Health in particular, rather than just research and academic institutions [65], so perhaps the time has finally come to integrate such behavioural parameter measurements into programmatically funded, longitudinal monitoring systems operating on national and regional scales [8].

## Abbreviations

LLINs: Long lasting insecticidal nets; IRS: Indoor residual spraying.

## Competing interests

The authors declare that they have no competing interests. The funders had no role in study design, analysis, decision to publish, or preparation of the manuscript.

## Authors’ contributions

GFK prepared the first draft of the manuscript based on several discussions amongst the authors, upon which AS, JEG, JS, CJD, and NC all provided detailed comments which contributed substantively to the content and conclusions of the final version. All authors read and approved the final manuscript.
